# Fast and cost-effective protocol to produce *Paracoccidioides* spp. antigens

**DOI:** 10.7705/biomedica.6874

**Published:** 2023-08-31

**Authors:** Karolina Rosa Fernandes-Beraldo, Roseli Santos de Freitas-Xavier, Adriana Pardini-Vicentini

**Affiliations:** 1 Laboratório Central, Universidade Federal de São Paulo, São Paulo, Brasil Universidade Federal de São Paulo Universidade Federal de São Paulo São Paulo Brazil; 2 Programa de Pós-Graduação em Ciências, Coordenadoria de Controle de Doenças, Secretaria de Estado da Saúde de São Paulo, São Paulo, Brasil Secretaria de Saúde do Estado de São Paulo Secretaria de Estado da Saúde de São Paulo São Paulo Brazil; 3 Laboratório de Micologia Médica (LIM53), Hospital das Clínicas, Faculdade de Medicina, Universidade de São Paulo, São Paulo, Brasil Universidade de São Paulo Universidade de São Paulo São Paulo Brazil; 4 Laboratório de Imunodiagnóstico das Micoses, Centro de Imunologia, Instituto Adolfo Lutz, São Paulo, Brasil Instituto Adolfo Lutz Instituto Adolfo Lutz São Paulo Brazil

**Keywords:** Paracoccidioides, paracoccidioidomycosis, antigens, immunologic tests, Paracoccidioides, paracoccidioidomicosis, antígenos, pruebas inmunológicas

## Abstract

**Introduction.:**

The existing methods for *Paracoccidioides* spp. antigen production are problematic in terms of standardization, specificity, stability, repeatability, and reproducibility.

**Objective.:**

To optimize the methodology for *Paracoccidioides* spp. antigen production and evaluate its applicability in paracoccidioidomycosis immunodiagnosis.

**Materials and methods.:**

The antigens were obtained from *Paracoccidioides* lutzii isolates (01, 66, and 8334), *Paracoccidioides* brasiliensis sensu stricto (113), and *Paracoccidioides* restripiensis (B-339). These fungi were grown at 36 °C ± 1 °C, on modified Fava-Netto agar, according to Freitas *et al*. (2018). *Paracoccidioides* lutzii antigens were obtained after 5, 10, and 20 days of culture, whereas *P. brasiliensis* and *P. restripiensis* antigens were obtained after 10 days. Antigens were evaluated in natura, 10 and 20 times concentrated. Antigenic capacity was evaluated using a double immunodiffusion assay against serum samples from patients with paracoccidioidomycosis, histoplasmosis, and aspergillosis, and random blood donors.

**Results.:**

Cross-reactivity between *Paracoccidioides* spp. antigens was observed when *P. brasiliensis*, *P. restrepiensis* antigens, and *P. lutzii* antigens were evaluated with the polyclonal antibodies against *P. lutzii* and *P. brasiliensis*, respectively. No cross-reactivity was obtained for polyclonal antibodies against Histoplasma capsulatum, Aspergillus fumigatus, and random blood donors. The proposed protocol allowed stable, repeatable, and reproducible genus-specific antigen production at a low cost and in a short cultivation time.

**Conclusion.:**

The proposed protocol allowed us to obtain genus-specific antigens that can be developed and reproduced in all laboratories in Brazil and South America, where paracoccidioidomycosis is a neglected disease, contributing to an early diagnosis, especially in endemic regions, regardless of the species.

## Resumen

**Introducción.:**

Los métodos existentes para la producción de los antígenos de *Paracoccidioides* spp. son problemáticos en su estandarización, especificidad, estabilidad, repetibilidad y reproducibilidad.

**Objetivo.:**

Optimizar la metodología para la producción de antígenos de *Paracoccidioides* spp. y evaluar su aplicabilidad en el inmunodiagnóstico de la paracoccidioidomicosis.

**Materiales y métodos.:**

Los antígenos se obtuvieron de aislamientos de P. lutzii (01, 66 y 8334), *P. brasiliensis* sensu stricto (113) y *P. restripiensis* (B-339). Estos hongos se cultivaron a 36 °C ± 1 °C en agar Fava-Netto modificado, según Freitas *et al*. (2018). Los antígenos de *P. lutzii* se obtuvieron a los 5, 10 y 20 días de cultivo y los antígenos de *P. brasiliensis* y *P. restripiensis* se obtuvieron a los 10 días. Los antígenos se evaluaron in natura, concentrados 10 y 20 veces. La capacidad antigénica se evaluó mediante un ensayo de inmunodifusión doble con muestras de suero de pacientes con paracoccidioidomicosis, histoplasmosis, aspergilosis y donantes de sangre aleatorios.

**Resultados.:**

Se observó reacción cruzada con *Paracoccidioides* spp. cuando se evaluaron los antígenos de *P. brasiliensis*, *P. restrepiensis* y *P. lutzii* frente a los anticuerpos policlonales contra *P. lutzii* y *P. brasiliensis*, respectivamente. No hubo reactividad cruzada con los anticuerpos policlonales contra Histoplasma capsulatum y Aspergillus fumigatus, ni contra los donantes de sangre aleatorios. El protocolo propuesto permitió la producción estable, repetible y reproducible de antígenos dirigidos de un género específico (Paracoccidiodes) en un tiempo corto de cultivo y a un menor costo.

**Conclusión.:**

El protocolo propuesto permitió obtener antígenos específicos de un género, que pueden ser desarrollados y reproducidos en todos los laboratorios de Antígenos de *Paracoccidioides* spp.: protocolo rápido Brasil y Surámerica donde la paracoccidioidomicosis es una enfermedad endémica y desatendida. Estos antígenos pueden contribuir al diagnóstico precoz de la infección, independientemente de la especie.

Paracoccidioidomycosis is the most important systemic mycosis in South America. It is caused by thermally dimorphic fungi belonging to the genera *Paracoccidioides*^1^^-^^3^. Approximately 80% of these cases were described in Brazil, followed by Colombia, Venezuela, and Argentina ^4^.

This mycosis presents a broad spectrum of clinical manifestations and occurs more frequently in males than females. The ratio is 1.7:1.0 among patients with the acute/subacute form and 22:1among those with the chronic form of the disease, affecting people aged 30 to 60 years, mostly in rural areas ^5^.

The definitive diagnosis of *Paracoccidioides* spp. infection is based on direct microscopic examination of biological specimens and their culturing, followed by macro-and microscopic observation for fungus identification ^2^^,^^6^. However, these procedures are insensitive and time-consuming, and it is difficult to obtain clinical specimens ^7^. In contrast, serological assays have played an important role in the early diagnosis of paracoccidioidomycosis and in monitoring its evolution and response to treatment ^6^^-^^9^. The standard serological assay involves double immunodiffusion; however, counterimmunoelectrophoresis, complement fixation, enzyme-linked immunosorbent assay (ELISA), immunoblotting, and dot ELISA may also be used ^2^^,^^6^^-^^9^.

The existing methods for producing *Paracoccidioides* spp. antigens are problematic in terms of standardization, specificity, stability, repeatability, and reproducibility ^10^^-^^12^. Thus, in this study, we sought to optimize the methodology for antigen production described by Freitas *et al*., evaluating its applicability in paracoccidioidomycosis immunodiagnosis using a double immunodiffusion assay ^13^.

## Material and methods

### Isolates of Paracoccidioides spp.

We seleted one *P. brasiliensis senso strictu* (S1-Pb 113), one *P. restrepiensis* (PS3-B-339), and three *P. lutzii* (01, 66, and 8334) isolates for this study. The isolates were cultured on Fava-Netto modified agar medium at 36 °C ± 1 °Cand were subcultured every 15 days.

### Preparation of Paracoccidioides spp. antigens

We employed the method described by Freitas *et al*. to prepare antigens from *Paracoccidioides* spp. isolates ^13^. Briefly, *P. lutzii* yeast cells were grown on Fava-Netto modified agar medium at 36 °C ± 1 °C for 5, 10, and 20 days. *Paracoccidioides brasiliensis senso strictu* and *P. restrepiensis* were grown in the same medium and temperature for seven days. Following the incubation period, 20 ml of a thimerosal-borate aqueous solution (1:5,000; Sigma Chemical Co. St. Louis, MO, USA) was added to each culture tube.

Culture tubes containing the fungal cells were incubated and submerged in a thimerosal-borate aqueous solution at room temperature for 24 hours. The supernatants were then aspirated using a sterile serological pipette and filtered through a sterile filter unit of 0.22 μm (Millipore Corporation, Billerica, MA, USA). The antigen solutions were concentrated 10-20-fold by lyophilization. After the protein dosage was determined using the Bradford method ^14^, the antigenic preparations were stored at -20 °C until use.

### Serum samples

We evaluated 24 serum samples from patients with proven paracoccidioidomycosis and previously reactive to *P. brasiliensis* antigens; ten serum samples from patients with serological reactivity, by double immunodiffusion, for Histoplasma capsulatum; ten serum samples from patients with serological reactivity, by double immunodiffusion for *Aspergillus fumigatus*, and nine serum samples from random blood donors. Paracoccidioidomycosis cases were classified according to the clinical practice guidelines for this disease ^3^. Anti-*P. brasiliensis* and anti-*P. lutzii* polyclonal antibodies obtained from rabbits were used as positive controls, and anti-Histoplasma capsulatum and anti-Aspergillus fumigatus polyclonal antibodies were used as negative controls.

The paracoccidioidomycosis serum samples evaluated in this study were obtained from patients residing in the state of São Paulo, a region endemic for *P. brasiliensis sensu stricto*. The serum samples evaluated in this study were stored at -20 °C in the biorepository of Laboratório de Imunodiagnóstico das Micoses, Instituto Adolfo Lutz, São Paulo, SP, Brasil.

### Ethics

The study was approved by the Internal Scientific Commissions and Bioethics in Human Research Committee of Instituto Adolfo Lutz (CEPIAL# 026/11).

### Double immunodiffusion assay

Double immunodiffusion assays were performed using the modified Ouchterlony method ^15^. The glass slides were covered with 3 ml of 1% agarose gel type II medium (Sigma Chemical Co., St. Louis, MO, USA) diluted in a buffered saline solution (pH 6.9) containing 0.4% sodium citrate and 7.5% glycine. Antigens (10 μl) were placed in the central well, while control and patient sera (10 μl) were added to the surrounding wells. The slides were incubated in a humid chamber at room temperature for 48 hours. Subsequently, they were washed with saline solution several times over a 24 hour-period. The gels were dried and stained in an ethanol-acetic acid-water mixture with 0.4% Coomassie brilliant blue R-250® (Sigma Chemical Co., St. Louis, MO, USA).

### Sodium dodecyl sulfate-polyacrylamide gel electrophoresis

Sodium dodecyl sulfate-polyacrylamide gel electrophoresis (SDS- PAGE) was performed as previously described by Laemmli (1970) ^16^. *Paracoccidioides* spp. antigens were diluted in a buffer containing 62 mM Tris- HCl (pH 6.8), 2% (w/v) SDS, 50 mM 2-mercaptoethanol, 10% glycerol, and 0.01% bromophenol blue; boiled for three minutes, and centrifuged before being seeded into the gels. The antigens were subjected to electrophoresis (20 mA at room temperature) in a 10% discontinuous SDS buffer system in a Mini-Protean II Electrophoresis Cell (Bio-Rad Laboratories, Hercules, CA, USA). The molecular mass was determined using a 10-250 kDa standard prestained protein marker (Bio-Rad Precision Plus Protein All Blue Standards, cat. # 161-037, Bio-Rad Laboratory, Hercules, CA, USA). The electrophoretic profile was visualized using silver nitrate (Sigma-Aldrich Co., St. Louis, MO, USA) stain, according to the protocol described by Ansorge (1985) ^17^.

### Polyclonal antibodies against Paracoccidioides spp. antigens

Two New Zealand white rabbits were immunized subcutaneously on the back with 100 μg of *P. brasiliensis* (Pb 113) and *P. lutzii* (Pb 01) antigens, emulsified (3:1 v/v) with complete Freund’s adjuvant (Sigma-Aldrich Co., St. Louis, MO, USA) in the first immunization, and with incomplete Freund’s adjuvant (Sigma-Aldrich Co., St. Louis, MO, USA) in the subsequent ones, repeated every week for four weeks. Rabbit sera were collected one week after the fourth immunization to assess the production of circulating antispecies-specific polyclonal antibodies using an immunodiffusion assay. One week after the booster dose, the rabbits were anesthetized intramuscularly with 10 mg/kg ketamine. The same protocol was used to obtain polyclonal antibodies against *H. capsulatum* and *A. fumigatus antigens*.

### Stability of Paracoccidioides spp. antigens

The stability of the antigens lyophilized and stored at -20^o^C was analyzed using a double immunodiffusion assay against species-specific polyclonal antibodies after three, six, and nine months.

### Screening of Paracoccidioides spp. antigens

Screening of *P. brasiliensis sensu strictu* (Pb 113), *P. restrepiensis* (B- 339), and *P. lutzii* (01, 66, and 8334) antigenic preparations was performed by determining the protein content, electrophoretic profile, and immunoreactivity against anti-*P. brasiliensis*, anti-*P. lutzii*, anti-*H. capsulatum*, and anti-*A. fumigatus* polyclonal antibodies and serum samples from random blood donors.

## Results

Using different cultivation times (5, 10, and 15 days) and antigen concentrations (*in natura*, 10- and 20-fold), we obtained 18 antigens from *P. lutzii*. After the evaluation of the protein content, electrophoretic profile, and immunoreactivity against polyclonal species-specific antibodies, three antigenic preparations were selected: antigen obtained from 01 isolate, cultured for 20 days and 10X (Th 01 20d 10X), antigen obtained from 66 isolate, cultured for five days, 20X (Th 66, 5d 20X), and antigen obtained from 8334 isolates cultured for 10 days, 20X (Th 8334 10 d 20X ) (table 1).

**Table 1:** Profile of the selected antigens

Antigens	Protein dosage (mg/mL)	Reactivity with C+ Pb	Reactivity with C+ Pl	Reactivity with C+ Hc	Reactivity with C+ Af
Th 01 20d 10X (FN)	71.09	R	R	NR	NR
Th 66 5d 20X (FN)	66.34	R	R	NR	NR
Th 8334 10d 20X (FN)	230.19	R	R	NR	NR
Th 113 7d 10X (FN)	99.58	R	R	NR	NR
Th 339 7d 10X (FN)	102.50	R	R	NR	NR

Th: Thimerosal; FN: Modified Fava-Netto agar; 10X: 10X concentrate; 20X: 20X concentrate; R: Reagent; NR: Non-reactive

C+Pb: policlonal antibody anti-antigen of P. *brasiliensis sensu strictu*

C+Pl: policlonal antibody anti-antigen of P. *lutzii*

C+Hc: policlonal antibody anti-antigen of H. *capsulatum*

C+Af: policlonal antibody anti-antigen of A. *fumigatus*

Four antigens from *P. brasiliensis sensu strictu* and *P. restrepiensis* were obtained, all produced after seven days of culture, following the laboratory’s previous experience with the isolates employed (113 and B-339). After evaluating the same parameters mentioned for *P. lutzii*, 10-fold concentrated antigens were selected (Th 113 7d 10X and Th 339 7d 10X ) (table 1).

**Figure 1:**
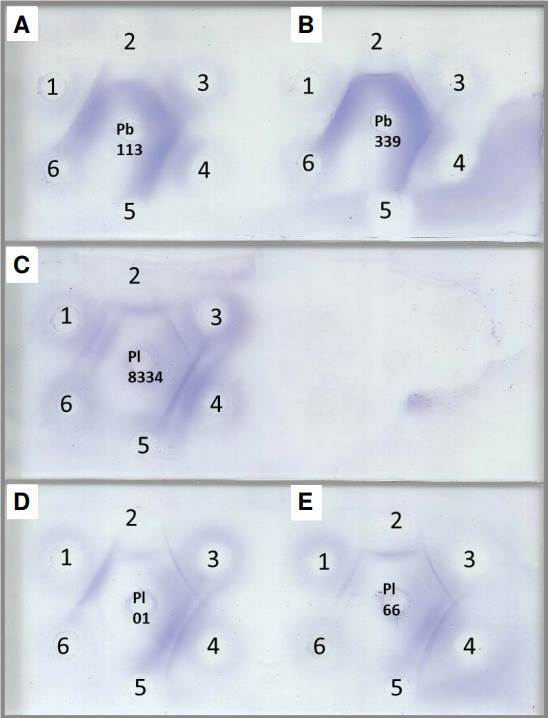
Double immunodiffusion assay with different *Paracoccidioides* spp. antigens. A) Th 113 7d 10X, B) Th 339 7d 10X, C) Th 8334 10d 20X, D) Th 01 20d 10X, and E) Th 66 5d 20X. 1) Anti-*P. lutzii* polyclonal antibody, 2) anti-*P. brasiliensis* polyclonal antibody, 3 and 4) paracoccidioidomycosis serum samples, 5) anti-*H. capsulatum* polyclonal antibody, and 6) anti-*A. fumigatus* polyclonal antibody.

Evaluation of antigenic preparations using double immunodiffusion assay demonstrated strong cross-reactivity when *P. brasiliensis sensu strictu* and *P. restrepiensis* antigens were evaluated against anti-*P. lutzii* polyclonal antibody; and when *P. lutzii* antigens were evaluated against the anti-*P. brasiliensis* polyclonal antibody (figure 1). Human serum samples with proven paracoccidioidomycosis also showed reactivity using double immunodiffusion assay against antigenic preparations of *P. lutzii*, reinforcing the desirable cross-reactivity between *Paracoccidioides* spp. and polyclonal antibodies. Out of the evaluated samples, 21 (87.5%) showed reactivity to Th 01 20d 10X antigen, 16 (66.66%) to Th 66 5d 20X antigen, and 20 (83.33%) to Th 8334 10d 20X antigen (figure 1 and table 2).

**Table 2:** Sensitivity and specificity of *Paracoccidioides* spp. antigens

Antigens	Reactivity with proven PCM	Sensitivity %	Specificity %
Th 01 20d 10X (FN)	21/24	87.5	85.29
Th 66 5d 20X (FN)	16/24	66.66	85.29
Th 8334 10d 20X (FN)	20/24	83.33	85.29
Th 113 7d 10X (FN)	24/24	100.00	100.00
Th 339 7d 10X (FN)	24/24	100.00	100.00

Th: Thimerosal; FN: Modified Fava-Netto agar; 10X: 10X concentrate; 20X: 20X concentrate;

PCM: Paracoccidioidomycosis

**Figure 2:**
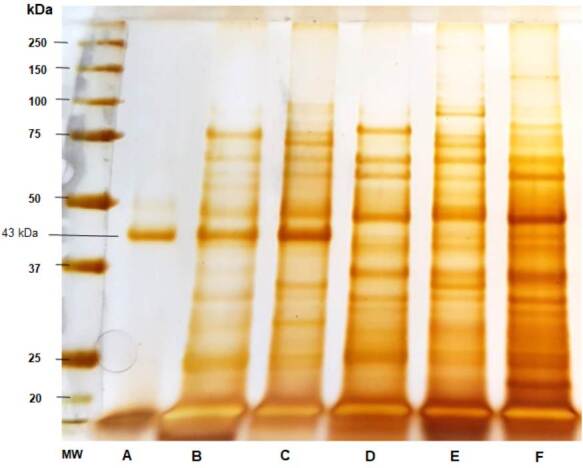
Sodium dodecyl sulfate-polyacrylamide gel electrophoresis (SDS-PAGE) of the *Paracoccidioides* spp. antigens. A) *Paracoccidioides brasiliensis* 113 culture-filtrate, B) Thimerosal 113 7d 10X, C) thimerosal B-339 7d 10X, D) Thimerosal 01 20d 10X, E) Thimerosal 66 5d 20X, and F) Thimerosal 8334 10d 20X.

There was no cross-reactivity between the *Paracoccidioides* antigens and the polyclonal antibodies anti-*H. capsulatum* and anti-*A. fumigatus*, neither with the random blood donors. However, we observed that three (3/10) serum samples from patients with histoplasmosis and two (2/10) from patients with aspergillosis showed cross-reactivity using double immunodiffusion assay when evaluated against *P. lutzii* antigens (Th01 20d 10X, Th 66 5d 20X, and Th 8334 10d 20X).

The stability results of the *Paracoccidioides* spp. antigens lyophilized and stored at -20 °C were analyzed after three, six, and nine months using double immunodiffusion assay against species-specific polyclonal antibodies. The results revealed that all the antigens retained their activity.

Analysis of the electrophoretic profile of the *Paracoccidioides* spp. antigens revealed several protein fractions with apparent molecular masses between 14 and 150 kDa. Some protein fractions were shared among *P. brasiliensis sensu strictu*, *P. restrepiensis*, and *P. lutzii* when obtained using the method described by Freitas *et al*. ^13^. Figure 2 shows gp43 production and secretion preferentially by B-339 and 113 *Paracoccidioides* spp. isolates (lanes A, B, and C). The culture-filtrate antigen of strain 113 predominantly comprised the fraction of gp43 (lane A).

## Discussion

Different antigens have been used for the immunodiagnosis of paracoccidioidomycosis using a double immunodiffusion assay. However, these antigen preparations were not standardized from one laboratory to another and included cytoplasmic extracts of yeast forms, concentrated filtrates, lyophilized filtrates, cell wall components, purified molecules, and recombinant antigens ^7^^,^^18^. These antigens were prepared from different *Paracoccidioides* spp. isolates, grown in different culture media and under different growth conditions ^12^^,^^18^. Besides, distinct *Paracoccidioides* spp. isolates can produce remarkably diverse qualities and quantities of antigens, a response that is also influenced by the culture medium ^11^^,^^18^.

Considering all the above in *Paracoccidioides* spp. antigen production, considerable disagreement regarding the sensitivity and specificity of the double immunodiffusion assay is not surprising. Vidal *et al*. compared the performance of laboratories from six medical mycology reference centers in Brazil that conducted routine serology with *P. brasiliensis* antigen preparations for the paracoccidioidomycosis diagnosis ^11^. The results showed inconsistencies among laboratories strong enough to result in conflicting information regarding patient management.

In addition to these methodological variations, in the last few years, false negative results have been reported in patients with proven paracoccidioidomycosis, especially in endemic regions of *P. lutzii*, suggesting antigen differences between species. Therefore, a comparative assessment of the antigen profiles of *P. brasiliensis sensu strictu*, *P. restrepiensis*, and *P. lutzii* is urgently needed. However, it must be emphasized that the protocol for obtaining these antigen preparations needs to be carefully considered because different parameters can alter the expression or secretion of protein fractions, both important for immunodiagnosis. According to the latter, this study developed a useful methodology for obtaining antigens from *P. brasiliensis* and *P. lutzii*, in contrast to the culture-filtered exoantigen, which, despite being indicated for the *complex P. brasiliensis*, does not show good results for *P. lutzii*^19^^,^^20^.

For several years, culture-filtrate preparations obtained from *P. restrepiensis* B-339 strain, rich in gp43, an immunodominant molecule of the species, was considered by several groups ^12^^,^^21^^,^^22^ as the “gold standard” antigen preparation, being distributed gracefully to different countries in South America like Colombia, Venezuela, and Argentina. However, Leitão *et al*. (2014) demonstrated that the gp43 of *P. lutzii* is produced in different amounts and has different epitopes according to the species ^23^. It strongly suggests that this molecule does not have the same serological importance between species.

These data confirm the need to reevaluate the parameters used to select antigen preparations to diagnose suspected cases. For example, it must be considered the migrating populations in Brazil. In addition, recent eco- epidemiological studies suggested that species can cohabit inthe same ecological niche ^24^^,^^25^. In this study, we observed cross-reactivity when *P. brasiliensis sensu strictu* and *P. restrepiensis* antigens were evaluated against anti-*P. lutzii* polyclonal antibody, and when *P. lutzii* antigens were evaluated against the anti-*P. brasiliensis* polyclonal antibody, suggesting that both species share protein and/or glycoprotein fractions.

Our results agree with those of Buccheri *et al*. (2018) ^26^ and Lenhard-Vidal *et al*. (2018) ^27^. Buccheri *et al*. (2018) described a patient with the acute form of paracoccidioidomycosis caused by *P. brasiliensis* with negative results on two reference centers’ routine screening for *P. brasiliensis* antibodies but positive for *P. lutzii* antigens implying that antibodies elicited during *P. brasiliensis* infection recognized the antigen fractions shared by both species ^25^. Lenhard-Vidal *et al*. (2018) showed that rabbit and human immune sera recognized several antigens from *P. lutzii* and *P. brasiliensis* complex isolates ^27^.

Regarding the three serum samples that did not show reactivity to *P. lutzii*, we must emphasize that other factors may contribute to the occurrence of false negative results, such as the clinical form of the disease, patient’s immunological status, and duration of antifungal therapy, all of which decrease the number of circulating antibodies and prevent their detection using serological methods, especially those with lower sensitivity ^28^. In such cases, immunoenzymatic assays, such as immunoblotting, can be used to improve the accuracy of immunodiagnostics. SDS-PAGE analysis of the antigen preparations revealed the presence of a wide variety of proteins in *Paracoccidiodes* spp. This method showed numerous protein and glycoprotein fractions in common for the three fungal species, corroborating the cross-reactivity observed in serological evaluations. It was also observed high gp43 secretion by *P. brasiliensis sensu stricto* and *P. restrepiensis*, with the majority being in the culture-filtered exoantigen. False negative results with this antigen preparation may be related to differences between species and the methods used to obtain the antigen preparations.

In this study, we evaluated the antigens obtained from three species of *Paracoccidioides*: *P. brasiliensis sensu stricto*, *P. restrepiensis*, and *P. lutzii*. Evaluation of the electrophoretic profile using SDS-PAGE revealed the presence of common protein fractions among different species, explaining the occurrence of cross-reactivity. Our results indicate the existence of genusspecific recognition, and we believe this characteristic is highly beneficial for the immunodiagnosis of paracoccidioidomycosis. In addition, we verified that using an antigen preparation composed of all the antigens obtained significantly increased the sensitivity of the agarose gel immunodiffusion assay (data not shown), thus contributing to not losing cases in serological evaluation.

Cross-reactivity between sera from patients with histoplasmosis or aspergillosis and *Paracoccidioides* spp. antigens has already been described in numerous studies and is related to antigen similarity between genera, translating it into a diagnostic problem, even when recombinant antigens are used in assays ^29^^,^^30^. Furthermore, overlapping ecological niches of different fungal species, cell wall composition, ubiquity of antigen determinants, and heterophile antibodies may contribute to cross-reactivity ^31^. Also, identified cross-reactivity may be influenced by memory antibodies or even rare co-infection by the two pathogens.

Dos Santos *et al*. (2015) evaluated latex assay performance using purified gp43 to identify circulating antibodies in serum samples from 65 patients with paracoccidioidomycosis. The specificity assay was determined using serum samples from 18 patients with histoplasmosis, 18 with aspergillosis, 13 with candidiasis, and 38 “healthy” individuals. The authors reported a sensitivity of 98.4% and a cross-reactivity of 16.6% for histoplasmosis, 11.11% for aspergillosis, and 7.7% for candidiasis.

In other study, the original 27 kDa recombinant protein was cloned and expressed in *Escherichia coli* to produce the 27 kDa molecular weight recombinant *Paracoccidioides* antigen. Indirect enzyme immunoassays were performed with the antigen against 160 human serum samples, of which 64 were from patients with *paracoccidioidomycosis*. The sensitivity mean was 73.4% compared with healthy individuals; specificity for patients with paracoccidioidomycosis was 87.5% but for patients with other fungal infections was 58.7%. These results showed high cross-reaction with the sera of patients infected with other mycoses ^32^^,^^33^.

Studies with a larger number of sera should be conducted. However, so far the preliminary evaluation of the antigen capacity by double immunodiffusion assay and the observed antigen profile by SDS-PAGE suggested a *Paracoccidioides* spp. antigen production with great potential for its use in the immunodiagnosis of patients with clinical suspicion of paracoccidioidomycosis.

This study proposes an alternative protocol for *Paracoccidioides* spp. antigen production. Among the advantages of the suggested methodology is the antigen obtention from *Paracoccidioides* spp. yeast stationary phase culture (without agitation). To dispense with a shaking incubator to obtain antigens results in savings for laboratories because this equipment costs an average of four to five times more than a conventional microbiological oven and requires more frequent corrective maintenance.

This protocol does not need an additional inactivation step of the fungal culture with thimerosal-borate solution (1:5,000), saving four days compared to obtaining culture filtrate. In this protocol, a thimerosal-borate aqueous solution (1:5,000) was used to extract and/or dissolve the soluble proteins (antigens) of *Paracoccidioides* spp. wall, for 24 hours. During that time, the yeasts were inactivated since this solution also has fungicidal and fungistatic functions, guaranteeing the safety of the obtained product.

In addition, most protocols aimed at obtaining culture filtrate recommend fungal culture for 20 days, culture inactivation, filtration on filter paper, and supernatant centrifugation in a refrigerated centrifuge (expensive equipment compared to a non-refrigerated centrifuge, which also requires a preventive maintenance contract). All of that to remove tiny fungal *Paracoccidioides* spp. cells, that escaped the filter paper, and thimerosal crystals.

In the proposed protocol, we obtained good results with antigens prepared after five days of culture (Th 66 5d 20X, *P. lutzii*) and seven days for *P. brasiliensis* and *P. restrepiensis* without the supernatant centrifugation and long filtering processes.

Finally, antigen concentration is often used to obtain culture-filtered antigens and other types of *Paracoccidioides* spp. antigens. Therefore, concentration does not make the process more expensive, as it is a common and necessary practice to obtain good antigen preparations. Although we used a lyophilizer, there are other economical and feasible methods for laboratories without this equipment ^22^.

We believe the advantages presented here support this simple, rapid, and accessible methodology for most mycoserology laboratories, particularly those with limited technological resources. The antigens obtained in this study have common antigen fractions among different species, allowing genus-specific recognition. Therefore, we think this protocol can be developed and reproduced in laboratories in Brazil and South America, where paracoccidioidomycosis is a neglected disease, contributing to an early diagnosis and favoring initiation of specific treatments, especially in endemic regions, regardless of the fungal species causing paracoccidioidomycosis.
